# Liposome-Assisted Drug Delivery in the Treatment of Multiple Sclerosis

**DOI:** 10.3390/molecules29194689

**Published:** 2024-10-03

**Authors:** Giuliana Greco, Maria Grazia Sarpietro

**Affiliations:** 1 Department of Biomedical and Biotechnological Sciences, University of Catania, 95123 Catania, Italy; giuliana.greco@phd.unict.it; 2 NANOMED-Research Center on Nanomedicine and Pharmaceutical Nanotechnology, University of Catania, 95125 Catania, Italy; 3 Department of Drug and Health Sciences, University of Catania, 95125 Catania, Italy

**Keywords:** multiple sclerosis, BBB, EAE models, nanocarriers, liposomes, drug delivery

## Abstract

Multiple sclerosis (MS) is an immune-mediated demyelinating disease of the nervous system that leads to neurological dysfunctions and severe disabilities. It is worth noting that conventional pharmacotherapy is poorly selective and causes toxicity problems and several systemic side effects. Thus, there is a need to develop new approaches to this medical challenge. The use of nanocarriers for drug delivery represents a good strategy to overcome several issues such as high therapeutic drug doses with side effects, such as diarrhea, nausea, and abdominal pain, and drug degradation processes; in addition, nanocarriers can provide controlled and targeted drug release. This review describes the application of liposomes for the delivery of pharmaceutical actives to target MS. Firstly, MS is explained. Then, liposomes are described along with their preparation, characterization, and stability. The literature about the use of liposomes for the treatment of MS is then analyzed.

## 1. Introduction

Liposomes, since their discovery in the 1960s by A.D. Bangham, have been widely used to encapsulate and deliver molecules to treat several diseases. Liposomes are mainly made of lipids and fatty acids that, being components of cell membranes, are considered biocompatible and biodegradable [1]. Liposomes can be composed of one or more lipid bilayers organized around an internal aqueous core, with the polar head groups oriented to the inner and outer aqueous phase and the hydrocarbon chains assembled within the hydrophobic interior [2]. This structure allows the liposomes to load and deliver molecules with different solubilities [3]. In addition, it allows for good interaction with cell membranes, promoting efficient cellular uptake.

Liposomal encapsulation of drugs reduces systemic toxicity and improves tolerable dose regimens for a variety of therapies. This review will focus on the application of liposomes in MS.

## 2. Multiple Sclerosis

### 2.1. Epidemiology

MS is an inflammatory, autoimmune, demyelinating neurodegenerative disease of the central nervous system (CNS). MS is characterized by two pathological hallmarks, inflammation with demyelination, which lead to axonal loss. According to data from the third edition of the *Atlas of Multiple Sclerosis* published by the Multiple Sclerosis International Federation (MSIF) [4], the prevalence of the pathology has increased over the last few decades, from 2.3 million people in 2013 to 2.8 million in 2020 and 2.9 million in 2023. The prevalence varies widely in different regions around the world, but Europe and America have reported the highest number of people affected, with 133 per 100,000 and 112 per 100,000, respectively, compared to Africa and South East Asia, where the prevalence is lower, with 5 people per 100,000 and 9 per 100,000 diagnosed with MS, respectively. MS is commonly diagnosed in young adults; indeed, the onset occurs in the range age of 20–40 years, although some cases are reported during childhood, called pediatric MS, and others occur in the group aged over 50 years. The percentage of females affected by MS (69%) is nearly twice that of males (31%); the reasons remain unclear, but it is probably due to hormonal and genetic differences, as well as environmental and social factors.

### 2.2. Etiology

**Figure 1:**
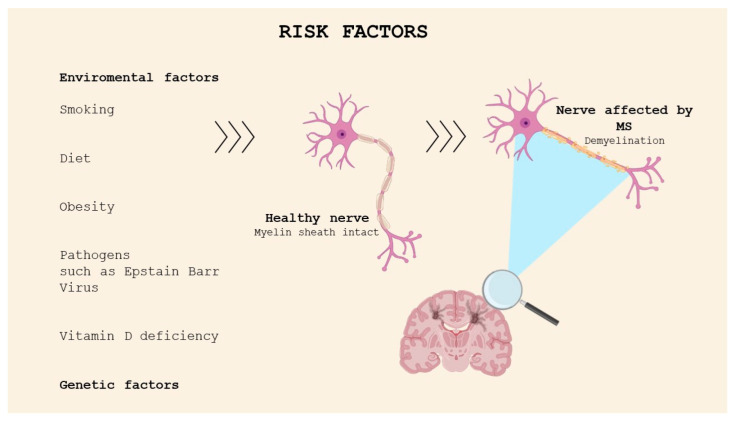
Risk factors for MS.

MS etiology is still unknown; however, it can be defined as a multifactorial disease resulting from a combination of environmental factors and genetic susceptibility (Figure 1). The environmental factors include exposure to viral and bacterial agents, especially Epstein Barr Virus (EBV), found in the brain of people with MS [5]. The data show that the risk of developing MS is higher in people infected with EBV in childhood or adolescence compared with non-infected individuals. A recent study was conducted in a cohort comprising more than 10 million young adults on active duty in the US military; 955 of them were diagnosed with MS during their period of service. It was found that the risk of MS increased 32-fold after infection with EBV but did not increase after infection with other viruses. The findings of this study suggested that EBV could be the leading cause of MS [6]. The mechanisms correlating the probability of onset of the pathology and EBV infection are not clear; molecular mimicry is historically a popular theory [7].

Low sunlight exposure and vitamin D deficiency have been identified as possible factors responsible for the increasing risk of developing MS. This has been investigated through several studies, which have reported increasing incidence of MS with latitude and the distance north or south of the equator; moreover, migration from high-risk areas to low-risk regions in childhood seems to reduce the risk of developing MS [8]. Indeed, the latitude is correlated with exposure to UVB sun rays, which stimulate cutaneous vitamin D production. The association between smoking, diet, stress, and MS has also been studied.

MS cannot be defined as a hereditary disease, but the familial clustering is determined by genetic factors. The results of twin and family studies have demonstrated that the risk rate in monozygotic twins, which have the same genetic heritage, is approximately 25%, while, in dizygotic twins and first-degree relatives, which share 50% of their genetic information, the percentage decreases to 2–5% [9]. This evidence confirms the higher risk of developing the pathology for family members of MS patients.

The association between MS and specific human leukocyte antigen (HLA) haplotypes was identified in early 1970 [10]. The HLA system is a group of genes on chromosome 6 acting as a major histocompatibility complex (MHC). The strongest genetic risk is represented by HLA-DRB1*1501 allele expression, but the genotypes HLA-DRB5*0101, HLA-DQA1*0102, and HLA-DQB2*0602 can be seen in all populations, except Sardinians and some Mediterranean groups [11]. Additional variations in groups of genes, such as T-cell receptor (TCR) genes, immunoglobulin (Ig) genes, or myelin basic protein (MBP) genes, contribute to the increasing risk of developing MS [12].

### 2.3. Clinical Course and Symptoms

**Figure 2:**
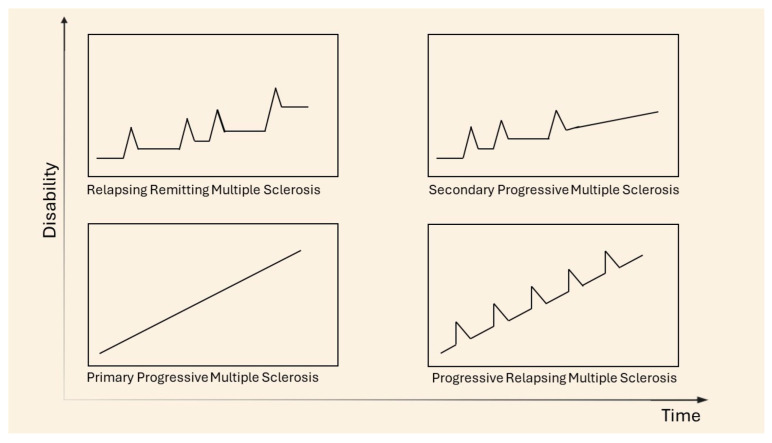
Clinical courses of MS. Adapted from [20].

In 1996, the U.S. National Multiple Sclerosis Society (NMSS) Advisory Committee on Clinical Trials in Multiple Sclerosis described four clinical courses for MS, later revised in 2013 and subsequently identified as subtypes, allowing for the classification of the disease, currently known as the Lublin classification. The main subtypes are distinguished as relapsing remitting MS (RRMS), secondary progressive MS (SPMS), primary progressive MS (PPMS), and progressive relapsing MS (PRMS) [13] (Figure 2). Clinically isolated syndrome (CIS) refers to an acute episode of inflammation or demyelination affecting one or more sites in the central nervous system, which may be considered the first neurological episode and could evolve into RRMS. Not all episodes of CIS progress to MS; however, 85% of diagnoses are preceded by at least one episode of CIS [14]. The most frequently diagnosed phenotype is relapsing remitting (85–90% of diagnoses), characterized by alternating phases of neurological dysfunctions, known as attack or relapse phases, followed by periods of remission. Typical signs and symptoms during the acute phase depend on the location of the demyelinating lesions, with the most common manifestations including optic neuritis, sensory and/or motor manifestations of myelitis, and brainstem symptoms such as internuclear ophthalmoplegia. The acute phase generally develops over hours to days, reaching a plateau that lasts several weeks before regressing into the remission phase. Around 65% of RRMS diagnoses may evolve into the secondary progressive form (SPMS) after 10–15 years, which is considered the second phase of the disease. In the SP form, the neurological disability progresses, becoming an irreversible condition where remission phases become less frequent [15]. The primary progressive form accounts for 5–15% of diagnoses and is characterized by the rapid progression of neurological symptoms from onset, without periods of remission. Recently, relapse-associated worsening (RAW) and progression independent of relapse activity (PIRA) have been introduced as mechanisms by which the irreversible progression of disability occurs. RAW is responsible for the permanent accumulation of disability in RRMS due to incomplete recovery from a relapse, while PIRA is considered to be the main source of symptoms worsening in the progressive forms of MS (primary and secondary progressive MS). Kappos et al., in a study in 2020, demonstrated that the disability accumulation in relapsing MS was not associated with overt relapses, challenging the current clinical distinction between relapsing and progressive forms of MS [16]. The most common symptoms in MS include sensory disturbances, such as paresthesia, dysesthesias, diplopia, and vertigo, which lead to compromised balance and mobility as well as weakness and stiffness. Optic neuritis is often one of the initial signs emerging at the onset of the disease and can result in vision loss. Trigeminal neuralgia and chronic neuropathic pain often occur in MS patients. Cognitive impairment, anxiety and depression, and other neuropsychiatric presentations impact the autonomy and quality of life of patients [17]. Progressive relapsing is the rarest form, characterized by the earliest onset of symptoms and the most rapid progression of the disease, overlapping with the primary progressive form. The classification of pathology has been undertaken with the aim of improving the accuracy of diagnoses, studying, and understanding the unique immunopathogenic profile and tailoring treatment [9,18,19].

### 2.4. Pathogenesis

MS pathogenesis remains unclear and complex; however, inflammatory and autoimmune components are the two most representative features of the disease. A well-established hypothesis involves the infiltration of autoreactive T cells into the central nervous system, crossing the BBB, which loses its integrity with increased permeability. Under physiological conditions, the body possesses a regulatory mechanism to control the activation of autoreactive cells in the periphery. However, scientific evidence suggests that in patients affected by MS, these regulatory cells, including CD4+, regulatory B cells, or NF cells, become dysfunctional, impairing the ability to control self-reactive cells. The activation of autoreactive T cells is probably due to the mechanism of molecular mimicry or failure of self-tolerance [21], with the response directed against one or more self-antigens, such as myelin basic protein (MPB) or proteolipid protein (PLP), the major constituents of the myelin sheath [22]. Autoreactive cells, in the periphery, overexpress adhesion molecules and specific receptors, allowing their penetration across the BBB, whose permeability increases due to cytokines, especially IL-17 and IL-22, released by Th17 cells. However, the pathogenicity of Th17 cells has been linked to the production of granulocyte-macrophage colony-stimulating factor (GM-CSF), a hematopoietic growth factor with several functions; in particular, it favors the survival and activation of myeloid cells and the differentiation of dendritic cells; it enhances antigen presentation; induces complement- and antibody-mediated phagocytosis; and mobilizes monocytes and other myeloid populations from the bone marrow to the bloodstream [23]. The infiltration of CD4+ and CD8+ T cells [24] prompts inflammation, attributable to the release of pro-inflammatory cytokines (INFϒ, IL-β, IL-17, IL-19, etc.) and chemokines, which promote the recruitment of other cells of the immune system, thus leading to a chronic condition. Centonze et al. studied the consequences of chronic inflammation in an experimental autoimmune encephalomyelitis (EAE) model, which results in synaptic degeneration occurring at an early stage of the disease, compromising nerve signal conduction [25]. The inflammatory state, persisting in MS patients, is responsible for damage to oligodendrocytes, a type of neuroglial cell, which produces the myelin sheath [26], causing irreversible axonal loss and myelin destruction. The pathological hallmark of MS is the formation of the demyelinating lesions or plaques, which involve both white matter and grey matter, including the cerebral or cerebellar cortex and brainstem nuclei. Recently, it has been suggested that B cells may also affect disease pathogenesis. It has been proposed that B cells could damage the CNS in MS through antibody-dependent and antibody-independent mechanisms. The functions of B cells in MS pathogenesis include antibody secretion by plasma blasts and plasma cells, antigen presentation to T cells and driving autoproliferation of brain-homing T cells, production of proinflammatory cytokines and chemokines, and the production of soluble toxic factors contributing to oligodendrocyte and neuronal injury. In addition, B cells contribute to the formation, in the meninges, of ectopic lymphoid aggregates, which may serve as a reservoir for Epstein Barr Virus infection [27]. These actions may contribute to both MS relapses and disease progression.

### 2.5. Animal Models of Multiple Sclerosis in Research

Experimental autoimmune encephalomyelitis (EAE) is the most widely used animal model for studying the pathogenic mechanisms and clinical features of MS, as well as for testing novel therapeutic strategies for the disease. EAE is induced through the inoculation of self-antigens, resulting in demyelination and axonal damage. Self-antigens have been inoculated in various animals, such as monkeys and guinea pigs, stimulating a T-cell-mediated immune response; however, genetically susceptible rats and mice have proven to be the most effective models [28]. The challenge was to reduce myelin basic protein (MBP), myelin oligodendrocyte (ODC), glycoprotein (MOG), proteolipid protein (PLP) to small encephalitogenic peptides. The inoculation of these different encephalitogenic peptides results in varied courses of pathology, e.g., PLP_139–151_ induces a relapsing–remitting (RR) form. Other self-antigens have been identified as playing a role in the autoimmune response of MS in the EAE model, including neurofascin NF 155, a myelin constituent, and several non-myelin constituents such as the neuronal membrane protein neurofascin NF186 and the neuronal cytoskeletal protein neurofilament-M.

In addition to the EAE model, the virally induced chronic demyelinating disease known as Theiler’s murine encephalomyelitis virus (TMEV) has been developed to provide a more comprehensive understanding of the pathology [29]. TMEV is another model used to replicate MS, induced in mice through viral infection, resulting in an immune response. It has demonstrated distinct features compared to EAE; indeed, mice are the exclusive animals used to induce inflammatory demyelinating disease, and the chronic progressive course of disease is the only replicable outcome.

The third model used to study MS is the toxic model; in this case, demyelination is caused through the administration of two agents represented by cuprizone and lysolecithin. This specific model reflects the process of de- and remyelination [30].

Animal models provide valuable information for studying the pathogenic mechanisms of MS and for testing therapeutic strategies; however, they have certain limitations. EAE is artificially induced by active or passive immunization, with symptoms appearing a few weeks post-immunization. In contrast, MS is not artificially induced and can remain latent for years, often being discovered only when evident symptoms appear. In EAE, demyelination primarily occurs in the spinal cord, whereas MS is a brain disease, with demyelination affecting the cerebral and cerebellar cortexes. EAE models are mainly based on inflammation, induced by autoreactive CD4+ T cells; however, several studies indicate that CD8+ T cells and B lymphocytes may play significant roles in propagating inflammation and tissue damage in MS. Regarding viral models, despite reflecting important characteristics of MS, they are challenging to use due to their complex pathogenesis; moreover, no specific viral infection linked to MS has been identified to date. The use of toxic models is limited to the study of the mechanisms of de- and remyelination [31].

### 2.6. Treatment

For many years, corticosteroids have been the only available therapy for the treatment of MS. Today, immunomodulatory and immunosuppressive therapies have been approved for the treatment of the pathology, allowing for a reduction in relapse frequency and the progression of disability. These are known as disease-modifying therapies (DMTs), as they have been demonstrated to modify the course of MS by acting through mechanisms which include the interference with cells of the immune system, e.g., sequestration of lymphocytes and shifts in cytokine secretion patterns. The first DMT approved (1993 by FDA) was Interferon-β 1b, which, together with Interferon-β 1a and glatiramer acetate, represents the oldest first-line injectable therapies in MS. Although these injectable therapies have been proven to be safe and effective, their use has diminished because of the development of alternative DMT, such as fumarates (dimethylfumarate, monomethylfumarate, diroximelfumarate), S1P-modulators (fingolimod, siponimod, ozanimod), and teriflunomide, which offer the advantage of oral administration, thus improving therapeutic adherence and patient compliance [32]. However, monoclonal antibody infusions belonging to the DMT class, such as natalizumab, ocrelizumab, ofatumumab, and alemtuzumab, are currently available treatments with higher efficacy than Interferon-β and glatiramer acetate [33,34]. Several studies have been carried out to encapsulate various DMT, such as dimethyl fumarate [35], fingolimod [36], and teriflunomide [37], in nanocarriers to improve efficacy and decrease side effects. There are two therapeutic approaches in the treatment of MS patients, the escalation approach, which starts with the administration of lower-risk and lower-efficacy DMT, followed by the administration of higher-risk and higher-efficacy molecules. The second approach, increasingly adopted in specialized MS centers, is highly effective and is based on the use of higher-efficacy DMT as a first-line treatment, just in the early stage of the pathology [38]. However, the first acute attack and the following relapses are treated with corticosteroids, generally in high doses and intravenously, for several days. Methylprednisolone and prednisolone are the two most common corticosteroids employed in the treatment of acute attacks [39]. In the late stages of the pathology, the patients are treated not only with chronic therapies such as DMT but also with symptomatic therapies.

## 3. Liposomes

Liposomes are lipid-based spherical-shaped vesicular systems, in which a lipophilic bilayer is sandwiched between two hydrophilic layers. The first description of swollen phospholipid systems was reported by Alec Bangham and colleagues in their 1965 publication [40]. These systems established the basis for model membrane systems [41]. Since then, a variety of enclosed phospholipid bilayer structures consisting of single bilayers, initially termed ‘bangosomes’ and then ‘liposomes’, have been described [42]. Gregory Gregoriadis established the concept that liposomes could entrap drugs and be used as drug delivery systems [43]. The versatility and advantages of liposomes as a drug delivery system for a variety of molecules with different activities are well studied and recognized. Liposomes fall into the general category of nanomedicines and play a key role in many diverse areas of health; they have been applied for the treatment of several diseases, such as cardiovascular, neurodegenerative, diabetes, cancer, and inflammation. Liposomes have been and continue to be used in the research and development fields; however, their use is limited because of certain problems. Several studies have been conducted to address some of these problems. For example, pharmacological challenges, such as destabilization by blood lipoproteins, uptake by the reticuloendothelial system, and rapid clearance from the blood circulation, have been addressed by modifying liposomes through pegylation. To increase the liposomes’ target specificity and the amount of therapeutic agent released at the site of disease, stimuli-sensitive and decorated liposomes have been designed. However, the transfer to large-scale production and clinical application of liposome formulations is hampered by some factors, such as instability, polydispersity, toxicity at repeated administration, and the capability of inducing immunostimulation and complement activation. New methods of preparation have been optimized to obtain liposomes with suitable size and distribution. The automation and control of the processes can assist in the pharmaceutical manufacturing and quality-assurance processes. To increase the success of new liposome formulations, dialogue between scientists, clinicians, and industry is indispensable [44].

### 3.1. Main Components of Liposomes

The main component of liposomes is glycerophospholipids, which are amphiphilic lipids made of a glycerol molecule bound to a phosphate group and to two fatty acid chains that may be saturated or unsaturated. The phosphate group is often bonded to another organic molecule. Depending on this organic moiety, natural phospholipids are classified as phosphatidic acid, phosphatidylcholine (PC), phosphatidylethanolamine (PE), phosphatidylinositol (PI), phosphatidylglycerol (PG), and phosphatidylserine (PS). Glycerophospholipids can be divided into two groups: natural and synthetic. The natural phospholipids most used to produce liposomes are PC and PE, which are abundant in plants and animals. Natural phospholipids are derived mainly from egg yolk and soya bean. Synthetic phospholipids are produced starting from natural lipids. Modifications of the natural lipids may occur in the head groups, in the aliphatic chains, and in the alcohols and, hence, a variety of synthetic phospholipids exist. Some phospholipids in synthetic form are 1,2-dimyristoyl-sn-glycero-3-phosphocholine (DMPC) 1,2-dipalmitoyl-sn-glycero-3-phosphocholine (DPPC) 1,2-distearoyl-sn-glycero-3-phosphocholine (DSPC), 1,2-dioleoyl-snglycero-3-phosphocholine (DOPC), 1,2-dioleoyl-snglycero-3-phosphoethanolamine (DOPE), 1,2-dipalmitoyl-sn-glycero-3-phosphoglycerol (DPPG), 1,2-distearoyl-sn-glycero-3-phosphoglycerol (DSPG), and 1,2-distearoyl-sn-glycero-3-phosphoethanolamine (DSPE) [45].

### 3.2. Classification of Liposomes

In an aqueous environment, phospholipids, due to their amphipathic character, have a strong ability to form stable bilayers. Hydrophobic chains are repelled by water molecules, and the self-assembly of liposomes into a closed bilayer occurs spontaneously.

**Figure 3:**
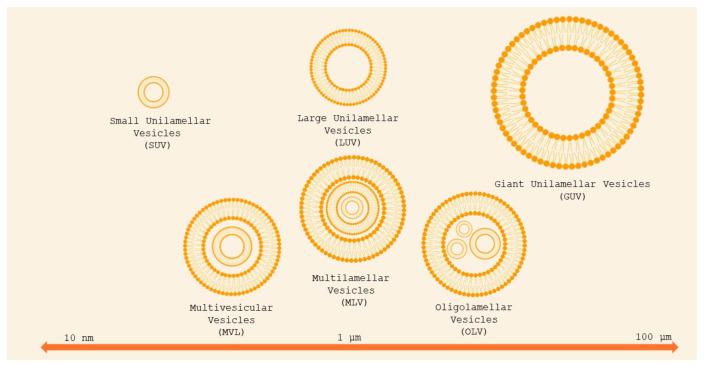
Classification of liposomes basing on size and lamellarity. Small unilamellar vesicles (SUVs) are less than 100 nm in diameter; large unilamellar vesicles (LUVs) are between 100 and 1000 nm; giant unilamellar vesicles (GUVs) are larger than 1 μm. Oligolamellar vesicles (OLVs) contain between two and five concentric bilayers; MLVs contain more than five concentric bilayers. Multivesicular vesicles (MVVs) encapsulate multiple non-concentric bilayer vesicles. Adapted from [46].

Liposomes can be classified as unilamellar vesicles (ULVs), which are particularly suitable for encapsulating hydrophilic compounds, oligolamellar vesicles (OLVs), multilamellar vesicles (MLVs), and multivesicular liposomes (MVLs), depending on the compartment structure and lamellarity (Figure 3) [3]. OLVs and MLVs show an onion-like structure, but OLVs present 2–5 concentric lipid bilayers, whereas MLVs contain more than five concentric lipid bilayers and are suitable for the encapsulation of lipophilic compounds. MVLs include hundreds of non-concentric aqueous compartments bounded by a single lipid bilayer and are used for the encapsulation of hydrophilic molecules [45]. Depending on the particle size, ULVs can be further divided into small unilamellar vesicles (SUVs, 30–100 nm), large unilamellar vesicles (LUVs, >100 nm), and giant unilamellar vesicles (GUVs, >1000 nm).

### 3.3. Production of Liposomes

There are several methods for the production of liposomes. In the following section, some of these methods will be explored [47].

#### 3.3.1. Thin-Film Hydration

This is the most common method employed for liposome production. In this method, lipids are solubilized in an organic solvent. The solvent is evaporated using a rotary evaporator under vacuum, leaving a thin film of lipids.

**Figure 4:**
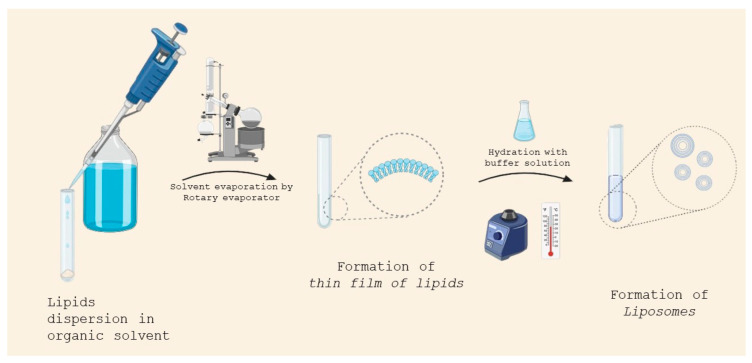
Liposome preparation by thin-film hydration.

The thin film is then hydrated in a buffer solution, whose temperature must be above the gel–liquid phase transition temperature of the lipid (Figure 4). If one or more hydrophilic drugs are desired to be encapsulated, they can be dissolved in the solution buffer before the hydration step, whereas lipophilic drugs can be dissolved in the organic solvent together with the lipid.

#### 3.3.2. Detergent Removal

In this method, lipids, amphiphilic molecules, and a surfactant (characterized by a high critical micelle concentration) are mixed in an organic solvent. The solvent is evaporated under low pressure, and a lipid film is formed. The lipid film is hydrated in an aqueous buffer, which leads to the formation of mixed micelles. The detergent is then removed to obtain LUVs. Several techniques are used for detergent removal: dialysis, size-exclusion chromatography, adsorption onto hydrophobic beads, or dilution [48,49].

#### 3.3.3. Injection

In this method, lipids are dissolved in an organic solvent, which can have a low or a high boiling point. In the case that a low-boiling-point solvent is used, the mixture is gradually injected into an aqueous solution containing the material to be encapsulated at 55 °C to 65 °C or under reduced pressure. In this way, the organic solvent is removed, leading to the formation of liposomes. This method has some disadvantages: the size of liposomes is heterogeneous (70 to 200 nm) and the compounds to be encapsulated are exposed to organic solvents at high temperature. If the organic solvent used has a higher boiling point, the mixture may be injected into an aqueous solution at room temperature under constant stirring, and the organic solvent can then be removed via dialysis or filtering. This method is most commonly used to prepare large volumes of liposomal formulations [50,51].

#### 3.3.4. Dehydration–Rehydration

This method permits the preparation of LUVs without the use of an organic solvent or detergents. The lipids and amphihilic molecules at low concentrations are directly dispersed into an aqueous solution, which is then subjected to sonication. The drug to be encapsulated may be added to the aqueous solution or mixed with the vesicles. When the water is let to evaporate, under nitrogen, liposomes form and entrap the drug molecules. A suitable amount of water is then added in order to form LUVs [52].

#### 3.3.5. pH Jumping

This method allows one to quickly prepare liposomes without the use of organic solvents. The method involves adding water to phosphatidic and phosphatidylcholine and subjecting it for a short time (<2 min) to a 3.5-fold increase in pH (from 3 to 10.5–11), resulting in the formation of liposomes.

The ratio of phosphatidic acid/phosphatidylcholine controls the percentage of SUVs and LUVs; generally, the higher the ratio of phosphatidic acid/phosphatidyl choline, the higher the percentage of SUVs obtained [53].

#### 3.3.6. Microfluidics

The microfluidic channel method has been recently proposed for the production of liposomes. This method involves the use of a tool where liquids flow within microscopic channels. Lipids are dissolved in ethanol or isopropanol, and the solution is injected (upright or in the opposite direction) into the aqueous medium within the microscopic channels. The continuous axial mixing of the organic and aqueous solutions leads to liposome formation. To avoid coagulation and separation, liposomes are stabilized with the addition of surfactants. This method permits control of the mixing process of organic and aqueous phases to obtain reproducible liposomes with an appropriate average size, polydispersity, morphology, and lamellarity [54].

### 3.4. Sizing of Lipid Suspension

Liposomes with a specific size can be obtained by submitting the formulation to sonication or to extrusion through membranes of well-defined pore sizes [55].

#### 3.4.1. Sonication

Sonication is one of the most used methods for the preparation of SUVs starting from MLVs. MLVs are sonicated either with a bath-type sonicator or a probe sonicator under a passive atmosphere. This method suffers from many disadvantages: very low internal volume/encapsulation efficacy, possible degradation of phospholipids and compounds to be encapsulated, titanium contamination from probe tip, and presence of MLVs.

In the probe sonication technique, the tip of a sonicator is directly immersed in the liposome dispersion. The energy input into dispersion is very high and can result in local heating; therefore, a water/ice bath must be used. In the bath sonication technique, the liposome dispersion in a beaker is placed into a bath sonicator. In this method, the temperature can be easily controlled, and an inert atmosphere can be used.

#### 3.4.2. Extrusion

In the extrusion technique, a preformed MLV suspension is forced through a polycarbonate filter with a defined pore size to yield particles (LUV or SUV) whose diameter is near the pore size of the filter used. Sometimes, the MLV suspension is pre-filtered through a filter of a larger pore size. This method permits the attainment of a homogeneous size distribution of the liposomes. The extrusion should be conducted at a temperature above the transition temperature of the lipid.

### 3.5. Targeting Strategies of Liposomes

Several strategies have been developed to enhance the targeted delivery of liposomal cargo to a specific site.

One passive approach to improve delivery to a specific organ site consists of prolonging the residence time of liposomes in the systemic circulation and, then, in preventing the macrophage uptake of liposomes. To achieve this, the surface of liposomes is modified with inert compounds, normally polymers, that enhance the steric stability of the liposomes and provide a barrier against opsonization. These surface-modified liposomes are referred to as stealth and circulate in the systemic circulation for a prolonged time, evading macrophage uptake. PEG-based conjugates are widely employed in the formulation of stealth liposomes [56]. Additionally, poly(2-methacryloyloxyethyl phosphorylcholine has been shown to increase the circulation time of liposomes [57].

Regarding the ligand-mediated strategy, the surface of liposomes is decorated with ligands, which bind to specific receptors. The uptake of the liposomes by target cells is initiated through ligand–receptor binding. Monoclonal antibodies, vitamins, proteins, peptides, carbohydrates, and DNA are widely used as they can selectively target cells that overexpress specific surface receptors [58].

More recently, to enhance both selectivity and efficacy in drug delivery to target sites, the surface of liposomes can be functionalized with a hydrophilic polymer and a targeting ligand. The targeting ligand can be directly conjugated to the surface and/or covalently bonded to the polymer chains [23].

Stimuli-responsive liposomes are systems capable of delivering and releasing drugs in a site-specific manner. These liposomes are designed to release the drug in response to specific stimuli, which can be endogenous, such as pH (the pH changes, inducing protonation/deprotonation of functional groups, can change the permeability of the liposomal membrane which induces morphological changes of the lipid bilayers) or enzymatic activity, which most often employs elevated enzyme expression to facilitate biochemical transformations that can activate drug release in specific microenvironments. External stimuli, such as light (where activating photons must safely penetrate biological tissues to initiate the release process), ultrasound waves, or heat, can also trigger drug release [59].

### 3.6. Liposome-Based Therapies

Doxil^®^, a polyethylene-glycol-coated doxorubicin liposome, was the first liposome-based drug formulation approved for human use in the USA (1995) for the treatment of ovarian cancer and AIDS-related Kaposi’s sarcoma [60]. Currently, there are several products on the market, with others in clinical development. Liposome-based therapies are approved for intravenous and intramuscular administration. The liposome structure allows one to encapsulate hydrophobic compounds in the lipid bilayer and hydrophilic compounds in the aqueous core. Due to their advantages, these nanocarriers have been used for the delivery of anticancer, antifungal, anti-inflammatory drugs as well as vaccines [61]. Examples of liposome-based therapies approved for antitumoral use include LipoDox^®^ and Myocet^®^, which are PEGylated and non-PEGylated liposomes, respectively, encapsulating doxorubicin; Onivyde^®^, a PEGylated liposome incorporating irinotecan; and Vyxeos^®^, consisting of cytarabine and daunorubicin. Other available products are Ambisome^®^ and Fungisome^®^, formulated to include amphotericin B, an antifungal drug. Liposomes have been used to develop vaccine formulations, such as Epaxal^®^ and Inflexal^®^ V, a hepatitis A vaccine, and COVID-19 mRNA-based vaccines. Today, more than 500 liposomal formulations, such as anticancer, analgesic, and immune modulators, are currently available in different clinical trial phases [45,62].

## 4. Liposomes in MS

### 4.1. Myelin Basic Protein (MBP) Liposomes

**Figure 5:**
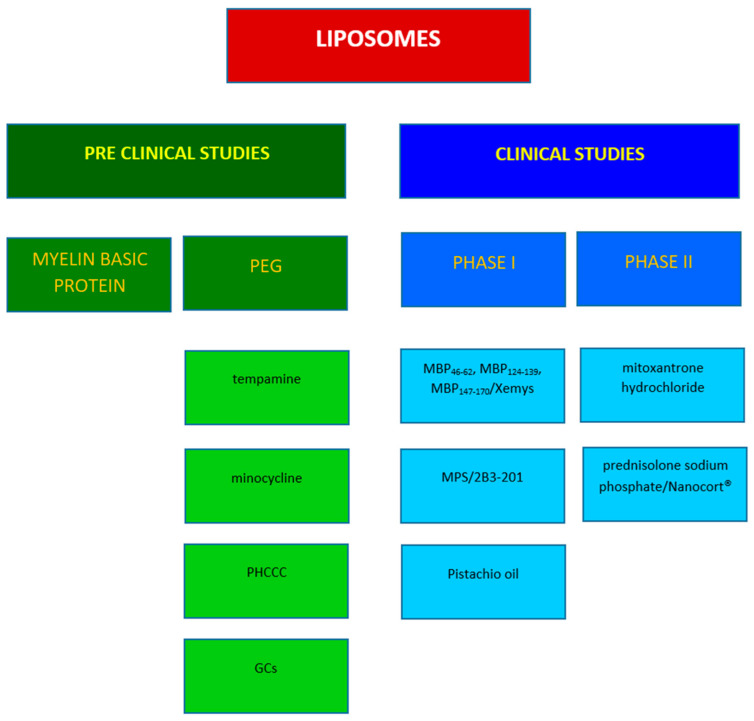
Representative studies in MS involving liposomes.

MS has been classified as an autoimmune disease. The autoreactive immune cells and T and B lymphocytes directed at myelin-related peptides have been thought to be responsible for the formation of inflammatory demyelinating lesions in the CNS of patients with MS [63], since they cross, together with macrophages, the blood–brain barrier (BBB). MBP, myelin oligodendrocyte glycoprotein (MOG), and proteolipid protein (PLP), the major constituents of the myelin sheath, have been linked with autoimmunity in the pathology and identified as autoantigens [64]. In recent decades, researchers have sequenced the immunogenic peptide epitopes within MBP, responsible for inducing the autoimmune response in MS patients; indeed, the administration of these fragments to animals results in the induction of EAE. However, encephalitogenic peptides have been used to prevent or suppress the EAE [65], probably due to their ability to induce immunotolerance, thus avoiding the immune response [66], since the team from the Weizzman Institute demonstrated that one of the MBP analogues, synthesized with the aim of inducing EAE, was able to suppress the disease. This was the rationale behind the design of glatiramer acetate (Copaxone^®^). Copaxone was used as the standard treatment in a study carried out through the administration of human MBP fragments via the nasal route in Dark Agouti (DA) rats after disease induction; compared to the other fragments, desirable results have emerged for MBP_46–62_, with a significant reduction in EAE [67]. However, liposomes were employed for the administration of MBP fragments, due to their guaranteed increased effectiveness. Strejan et al. compared the administration of MBP-liposomes and MBP in solution, via intravenous [68] and intracardiac routes [69], and they obtained a more effective reduction in clinical signs of EAE with the MBP-liposome formulation in both studies, highlighting the significance of liposomal nanocarriers as vehicles for active compounds. Avrilionis and Boggs explored the ability of the liposomal formulation bounding MBP fragments to reduce or suppress EAE in guinea pigs. They chose to inject both soluble MBP and liposomal-MBP via intraperitoneal and subcutaneous routes, and they achieved desirable results for liposomal peptides administered intraperitoneally, likely as a result of reduced degradation [70]. According to the literature [71], this observation has confirmed the protective role of liposomes against metabolic reactions; indeed, they prevent the enzymatic degradation of drugs and improve stability. Belogurov Jr. et al. encapsulated MPB immunodominant fragments into mannosylated liposomes and tested the therapeutic potential of the formulation in DA rats with induced EAE. The mannosylation of liposomes seemed to improve brain delivery [72]; this is probably correlated with the high affinity of mannose for glucose transporters (GLUTs), which are expressed at the BBB. They studied the activity of three different MPB fragments [67], injected subcutaneously as such or encapsulated in mannosylated or nonmannosylated liposomes in EAE DA rats. The best results, in terms of efficacy, were obtained from the administration of liposomal formulations; no effect emerged from the administration of MBP fragments as such, and mannosylated liposomal formulations were more effective than non-mannosylated MBP liposomes [73]. Belogurov Jr.’s group conducted clinical studies of MBP encapsulated in mannosylated liposome (Xemys); they evaluated cytokine levels [74] and tolerability [75] after Xemys administration, obtaining desirable results, in support of the hypothesis that Xemys could be used as a treatment for RRMS. The peptide fragments have been used not only as encapsulated compounds but also as functionalized elements, as in a study followed up by Shimizu et al. [76]. They used a model of primary progressive MS in mice induced by MOG; the model was treated with MOG-modified liposomes encapsulating doxorubicin. The therapeutic effect was compared with other formulations, such as functionalized empty liposomes, non-modified liposomes encapsulating doxorubicin, or free doxorubicin. Desirable results were obtained with MOG-modified liposomes encapsulating doxorubicin, while the other formulations failed (Table 1) (Figure 5).

### 4.2. PEGylated Liposome Encapsulating Glucocorticoids

Stealth liposomes, or long circulating liposomes, represent a second-generation class of liposomes obtained through the modification of the carrier’s surface, which is covered with water-soluble polymers, often including polyethylene glycol (PEG). Functionalization with PEG offers several significant advantages, rendering liposomes suitable for the treatment of various diseases, such as MS. PEG reduces adhesion to opsonic plasma proteins, thus avoiding the immune response and rapid elimination from the systemic circulation, consequently leading to an extension of plasma half-life [95]. Moreover, PEGylated liposomes exhibit a high affinity for inflamed tissues [96]. The treatment of the first and subsequent acute attacks in MS typically involves the administration of glucocorticoids (GCs) in high doses intravenously. This approach is based on their anti-inflammatory and immunosuppressive activities. Prednisolone, methylprednisolone, and dexamethasone are among the most frequently employed GCs. The limitation associated with their administration is their poor pharmacokinetics, characterized by an initial plasma peak followed by rapid clearance; thus, drug delivery systems such as liposomes have been employed to improve their pharmacokinetic properties. In a study conducted by Schmidt et al. [77], prednisolone phosphate was encapsulated into PEG-coated liposomes (prednisolone liposomes, PL) and administered to EAE rats. PL decreased the production of TNF-α, causing an amelioration of disease score, and it has been shown that two injections of liposomal prednisolone are superior to two injections of the free form of prednisolone at a five-fold higher dose.

Palmitoyl prednisolone was completely incorporated within PEGylated liposomes and tested on rats following intravenous administration; the effect of PEGylated liposomes was compared with that of non-PEGylated liposomes [78]. Pharmacokinetic studies have confirmed that liposomal palmitoyl prednisolone maintained a significantly higher concentration in the bloodstream than the non-PEGylated formulation. Other comparative studies have been conducted between PEGylated liposomal formulations and free active compounds, aimed at underlining the influence of liposomes on the half-life of vehiculated active compounds and the importance of using this type of drug delivery system. Linker et al. compared liposomal prednisolone (PL), liposomal methylprednisolone (MPL), and free methylprednisolone (MP), which is typically used for pulse therapy; a higher effect was demonstrated for MPL than for PL and the free form of GCs, both in acute and chronic models of MS [79]. Similarly, Schweingruber et al. confirmed the superior efficacy of the liposomal formulation over the free form of GCs through their study, which involved intraperitoneal administration to EAE mice models of two fluorinated GCs and two non-halogenated GCs [80]. The fluorinated compounds comprised dexamethasone and triamcinolone, whereas the non-halogenated ones included prednisolone and methylprednisolone. A comparative study was conducted between the fluorinated and non-halogenated compounds, resulting in the superior therapeutic efficacy of the former. However, the study focused on the encapsulation of GCs, which enhanced efficacy and altered the specificity and the mechanism of action.

Similar studies were performed by Gaillard et al. [81], where the efficacy of glutathione (GSH) PEG-covered liposomes encapsulating methylprednisolone, referred to as targeted liposome, was examined. GSH is an endogenous tripeptide that plays an important role in the process of functionalizing liposomes; indeed, it has been shown that GSH improves the efficiency of transcellular liposome transport across the BBB, enhancing targeted brain delivery through improved cellular uptake [97]. Non-targeted liposomal methylprednisolone, non-functionalized-GSH liposomes, and free form of methylprednisolone were employed as control treatment. Pharmacokinetic analysis and studies on brain uptake in rats revealed superior outcomes for the GSH-PEG liposomal methylprednisolone formulation in comparison to the non-targeted liposomal methylprednisolone and the free form of methylprednisolone.

This research was further expanded by Lee et al., who assessed the efficacy of the GSH-PEG-liposomal methylprednisolone formulation called 2B3-201 in MOG-EAE mice. Three pivotal parameters were considered, namely, the attenuation of clinical symptoms of the pathology, a reduction in inflammatory infiltration, and amelioration of tissue damage. In these respects, 2B3-201 demonstrated ten-fold higher efficacy than the free-form methylprednisolone [82].

Kanhai et al. conducted a first-in-human study regarding 2B3-201 to assess the safety, pharmacokinetics, and pharmacodynamics of the formulation. The study was conducted in two parts: the first was a double-blind, three-way crossover study in 18 healthy male subjects comparing ascending doses of 2B3-201, the free form of MP (active comparator), and a placebo; the second part of the study was open-label and included 18 healthy male subjects and 6 healthy female subjects. Subsequently, the study was extended to other healthy subjects, reaching a total of 46 healthy subjects. Based on pharmacokinetic properties, 2B3-201 proved to be a slow-release product, with a terminal half-life ten-times longer than MP. The slow release resulted in a significantly higher serum concentration of MP, which led to important pharmacodynamic properties, such as a marked reduction in lymphocyte levels, osteocalcin, and adrenocorticotropic hormone, compared to free MP. These promising results support the possibility of administering 2B3-201 as an alternative to free MP [83]. The potential advantages include a reduction in the numbers of hospital visits and a decrease in related healthcare costs; moreover, a reduction in side effects was caused by a high dose of MP. Another clinical trial was carried out comparing the intravenous administration of PEG-liposomal prednisolone sodium phosphate (Nanocort^®^) and MP (SoluMedrol^®^) to assess the efficacy and safety of the former. The study was randomized, international, multicenter, and phase II, recruiting patients with acute exacerbation of RRMS or patients with CIS. The study was terminated, and no results were published [98] (NCT01039103) (Table 2).

An interesting study was conducted by Avnir et al., in which long circulating liposomes were used to encapsulate methylprednisolone [84] and were tested in the acute EAE mouse model through a comparison between the free form of the active compound and conventional therapies for MS. They demonstrated the superior efficacy of methylprednisolone compared to Copaxone and betaferon.

Long circulating liposomes were also used to encapsulate dexamethasone phosphate (LCL-DXP) [85], and free DXP, LCL-DXP, and PBS (as negative control) were compared and administered in EAE female mice. The free DXP and PBS did not demonstrate an effect on the acute or chronic phases of the disease, while LCL-DXP proved to be effective in the acute phase and resulted in a reduction in disease activity. This study was highly intricate; indeed, another group of mice with chronic DSS-induced colitis, a model of Crohn’s disease (CD), underwent the same treatment. From this comparative study, it emerged that LCL-DXP caused a shift in the macrophage population from M1 to M2, which in the CD model exacerbated the pathology, whereas in the MS model, it not only confirmed its effectiveness but also elucidated the mechanism of action (Table 1) (Figure 5).

### 4.3. PEGylated Liposomes Encapsulating Other Molecules

Long circulating liposomes have been employed to encapsulate various molecules, among which is tempamine (TMP). TMP is a potent radical scavenger with potential applications in the treatment of neurodegenerative diseases, which are always characterized by free radical production. TMP was administered intravenously as a solution to mice. This treatment showed no effect on EAE. In contrast, the formulation of PEGylated liposomal TMP decreased clinical symptoms and disease duration [86]. Turjeman et al. encapsulated TMN and methylprednisolone hemisuccinate (MPS) within nano-sterically stabilized liposomes made of various phospholipid types, such as DMPC, DPPC, egg phosphatidylcholine, and cholesterol. They conducted tests in the acute EAE mouse model. The liposomes loaded with TMN and MPS demonstrated superior efficacy compared to the negative control (saline). Additionally, egg phosphatidylcholine-based liposomes loaded with TMN exhibited increased brain penetration when compared to the free form of TMN, which was rapidly cleared [87].

Liposomal PEGylated formulations also offer an advantage in the administration of minocycline, an oral tetracycline derivative currently under investigation for the treatment of neurodegenerative disorders, including MS [88]. PEG minocycline liposomes were administered intravenously to EAE mice, and it was found that a lower dose is sufficient compared to free minocycline; this could also potentially reduce side effects associated with its administration.

PHCCC is a positive allosteric modulator of metabotropic glutamate receptor 4 (mGluR4), known for promoting a shift from an inflammatory cytokine profile, secreted by dendritic cells, to cytokines promoting Treg, responsible for an anti-inflammatory response. Due to its toxicity and short half-life, PHCCC was encapsulated into PEGylated liposomes. The liposomes were formulated with different PEG percentages, and an increase in PHCCC loading levels was observed for higher PEG percentages. This confirmed the important role of PEG in the functionalization of liposomes, as it is responsible for the increased circulation time. The formulation was tested on primary dendritic cells, and a reduction in pro-inflammatory cytokines emerged; moreover, the toxicity of PHCCC-liposomes was four-fold less compared to the free PHCCC [89].

Several studies have demonstrated a deficiency in essential polyunsaturated fatty acids (PUFAs) in MS patients; however, the cause remains unclear and may involve metabolic and nutritional alterations [100,101]. PEGylated nanoliposomes of pistachio unsaturated oils (PEGNLPUOs) were formulated by Jebali et al. using an extracted oily mixture of *Pistacia vera* L. rich in PUFAs, Omega-3, Omega-6, and also iron, calcium, copper, and other minerals [102]. The effect of the formulation (10% *v/v* pistachio oil) on the inflammatory response was tested in an EAE mouse model, revealing desirable results such as a reduction in Th1 and an increase in Th2 cells, leading to improved disease symptoms. In light of the promising results from the preclinical studies, researchers proceeded with a human study to assess the therapeutic effect of PEGNLPUOs and their efficacy in attenuating inflammation in RRMS. The study was a phase I, randomized, double-blind, placebo controlled clinical trial involving 50 woman with MS. Oral administration of PEGNLPUOs resulted in a Th2-biased immune response, improved clinical outcomes, and enhanced quality of life in MS patients [99]. Another clinical trial was conducted at the Xuanwu Hospital Capital Medical University (China) to evaluate the efficacy and safety of a liposomal intravenous formulation of mitoxantrone hydrochloride. It was a phase II, multicenter, randomized, single-arm, open-label clinical study. Among the criteria used to recruit participants, a diagnosis of RRMS and SPMS was necessary. Subjects were divided into three groups and treated with 4 mg/m^2^, 8 mg/m^2^ and 12 mg/m^2^, respectively, of mitoxantrone hydrochloride liposomes, with the aim of obtaining, as a primary outcome, the cumulative number of new lesions at the end of 48 weeks of treatment in the brain. No results have been posted; however, the application and administration of liposomal formulations in humans could suggest the potential advantages they may offer to patients [data available at Clinicaltrial.gov; accessed on 18 September 2024; NCT05496894] (Table 1 and Table 2, Figure 5).

### 4.4. Diagnostic Role of Liposomes in the MS

In recent years, liposomes have played a significant role in the context of MS, both as carriers and as tools for studying the immune response in patients. The etiology of MS has not been fully elucidated yet; however, the autoimmune component represents one of the primary elements analyzed for a comprehensive understanding of the condition. For this purpose, several research groups have employed liposomes loaded with lipid antigens, such as gangliosides, aiming to identify the immune response against them. Gangliosides are a class of acidic glycosphingolipids normally found in the nervous system as a major constituent of human myelin [103]; nevertheless, several studies have demonstrated an increase in antibodies against gangliosides in the serum of MS patients. This observation has led to the hypothesis involving gangliosides as antigens in the autoimmune response.

Arnon et al. prepared liposomes containing gangliosides as haptens within their lipid bilayer and tested them against serum from both MS patients and controls. A considerable complement-dependent lysis of the liposomes was observed in the serum of MS patients, in contrast to the results from the serum of normal patients. This indicated the presence of antibodies in the serum of MS patients capable of lysing liposomes due to the presence of gangliosides, identified as autoantigens [90]. The same principle was applied by the Feix research group; however, they employed a specific technique known as the spin-membrane immune assay (SMIA), which detected complement-mediated membrane lysis using liposomes containing gangliosides and a marker for lysis. Similarly, a distinct behavior was observed between the serum of MS patients and that of healthy individuals. Specifically, a higher value of released marker was noted in MS patients compared to the negative control. These data suggested that complement-mediated lysis of ganglioside-containing liposomes resulted in the release of the encapsulated marker due to the presence of antibodies against gangliosides in the serum of MS patients [91].

Mullin et al. devised liposomes containing both G_M1_, a specific ganglioside, and radiolabeled carbon 14 glucose within their aqueous compartment and tested them on the serum of MS patients. The study relied on quantifying ^14^C glucose in the serum to identify the presence of antibodies directed against G_M1_. The release of ^14^C glucose in the serum of MS patients was higher than in controls, suggesting the presence of anti-G_M1_ antibodies [92].

Another research group also identified the presence of antibodies against G_M1_ in the serum of MS patients by applying the Liposomes Lysis Assay [93].

The assessment of rubidium isotope release from the aqueous compartment of liposomes encapsulating galactocerebroside represents another assay derived from Slovick et al. for the swift detection of antibodies against galactocerebroside. Liposomes incorporating galactocerebroside and rubidium have been tested using serum from MS models. After a straightforward filtration step, the amount of rubidium isotope released from residual liposomes can be determined [94].

## 5. Conclusions

MS is a complex pathology whose treatment is complicated by the presence of physiological barriers, which hinder the entry of drugs into the central nervous system. Liposomes have gained significant attention as a drug delivery system for numerous types of drugs and represent a promising approach for the development of new targeted therapies in MS. They are easily created and can also be loaded with more than one molecule that can target various aspects of a complex disease like MS. Several studies have demonstrated the successful administration and promising results of liposomes in animal models. The great potential for improving liposomal formulations with appropriate characteristics and functionalization creates future perspectives for the enhancement of current MS therapies.

## Figures and Tables

**Table 1:** Summary of liposomal formulation studies.

	Drug	Liposome Composition	Administration Route	Reference
TREATMENT	prednisolone phosphate	DPPC, DSPE-PEG 2000, cholesterol	injection into a tail vein (female Lewis rats)	[77]
	palmitoyl prednisolone	DSPC, Cholesterol, DSPE-PEG 2000	injection into the jugular vein (male Wistar rats)	[78]
	methylprednisolone	DPPC, DSPE-PEG 2000, cholesterol	injection in the tail vein (Lewis rats)	[79]
	methylprednisolone-21-hydrogensuccinat, prednisolone-21-hydrogensuccinat, triamcinolone-acetonyd-21-hydrogen-phosphate, dexamethasone-dihydrogen-phosphate	DPPC, DSPE-PEG 2000, cholesterol	i.v. or i.p. injection (C57BL/6 mice)	[80]
	methylprednisolone	l-α-phosphatidylcholine, hydrogenated (Soy), cholesterol, mPEG-DSPE, reduced GSH	i.v. injection (male Lewis rats)	[81]
	MPS	l-α-phosphatidylcholine, hydrogenated (Soy), cholesterol, mPEG-DSPE, reduced GSH	i.v. injection (female C57BL6/J mice)	[82]
	MPS	l-α-phosphatidylcholine, hydrogenated (Soy), cholesterol, mPEG-DSPE, reduced GSH	infusion (first in-human study)	[83]
	methylprednisolone succinate sodium salt,	Hydrogenated soybean phosphatidylcholine, POPC, DSPC, cholesterol, DSPE-PEG 2000	injection (female SJL/J, C57Bl/6 and BALB/c mice)	[84]
	dexamethasone phosphate	DSPE-PEG 2000, DPPC, cholesterol	i.v. (female SJL mice)	[85]
	TMP	Egg phosphatidylcholine, Hydrogenated soybean phosphatidylcholine, DSPE-PEG 2000, cholesterol	i.v. (C57Bl/6J female mice)	[86]
	TMP, methylprednisolone	Egg phosphatidylcholine, hydrogenated soybean phosphatidylcholine, DMPC, DPPC, cholesterol, DSPE-PEG 2000	i.v. (SJL/J mice)	[87]
	minocycline	DPPC, DSPE-PEG 2000, cholesterol	i.v. (Female C57BL/6 mice)	[88]
	PHCCC	cholesterol, DOPC, DSPE-PEG 2000	in vitro study (primary Dendritic cells isolated from the spleens of female C57BL/6 J mice)	[89]
	**Molecule**			
DIAGNOSIS	GM1, GM 2, GM 4, galactocerebroside	phosphatidyl choline, cholesterol, dicetyl phosphate, α-tocoperol, 1-aminonapththalene-3,6,8-trisulfonic acid (fluorphore), bispyridinium xylene (quencher)	human serum, cerebrospinal fluid (from MS patients)	[90]
	GM1	DMPC, cholesterol, dicetyl phosphate	human serum (from MS patients)	[91]
	GM1	DMPC, cholesterol, dicetyl phosphate, [14C]glucose	human serum (from MS patients)	[92]
	GM1, GM4, GD1A, GD1B	sphingomyelin, dicetylphosphate, cholesterol, glycolipids	human serum (from MS patients)	[93]
	bovine brain galactocerebroside	dipalmitoyl lecithin, dicetyl phosphate, Cholesterol, [^3^H]cholesterol, ^86^Rb	blood (from human donor)	[94]

DMPC = 1,2-dimyristoyl-sn-glycero-3-phosphocholine; DOPC = 1,2-dioleoyl-sn glycero-3-phosphocholine; DPPC = 1,2-dipalmitoyl-sn glycerol-3-phosphocholine; DSPC = 1,2-distearoyl-sn-glycero-3-phosphocholine; DSPE-PEG 2000 = 1,2-distearoyl-sn-glycero-3-phosphoethanolamine-*N*-[methoxy(polyethylene glycol)-2000]; MPS = methylprednisolone hemisuccinate; PHCCC = *N*-Phenyl-7-(hydroxy-imino)cyclopropa[*b*]chromen-1*a*-carboxamide; POPC = 1-Palmitoyl-2-oleoyl-sn-glycero-3-phosphocholine; TMP = tempamine.

**Table 2:** Liposomal formulations in clinical studies.

Drug/Name	Conditions	Phase	Reference/NCT Number
MBP_46–62_, MBP_124–139_, MBP_147–170_/Xemys	RRMS, SPMS	I	[74]
MBP_46–62_, MBP_124–139_, MBP_147–170_/Xemys	RRMS, SPMS	I	[75]
MPS/2B3-201	MS	I	NCT02048358
Pistachio oil	MS	I	[99]
prednisolone sodium phosphate/Nanocort^®^	Acute exacerbation of RRMS, CIS	II	NCT01039103
mitoxantrone hydrochloride	RMS	II	NCT05496894

CIS = clinically isolated syndrome; MBP = myelin basic protein; MPS = methylprednisolone hemisuccinate; RMS = relapsing multiple sclerosis; RRMS = relapsing remitting multiple sclerosis.

## Abbreviations

BBB	blood–brain barrier;
CIS	clinically isolated syndrome;
CNS	central nervous system;
DA	dark agouti;
DMPC	1,2-dimyristoyl-sn-glycero-3-phosphocholine;
DMT	disease modifying therapies;
DOPC	1,2-dioleoyl-sn glycero-3-phosphocholine;
DPPC	1,2-dipalmitoyl-sn glycerol-3-phosphocholine;
DSPC	1,2-distearoyl-sn-glycero-3-phosphocholine;
DSPE	1,2-distearoyl-sn-glycero-3-phosphoethanolamine;
DSPE-PEG 2000	1,2-distearoyl-sn-glycero-3-phosphoethanolamine-*N*-[methoxy(polyethylene glycol)-2000];
EAE	experimental autoimmune encephalomyelitis;
EBB	Epstein Barr Virus;
GCs	glucocorticoids;
GSH	glutathione;
GUVs	giant unilamellar vesicles;
HLA	human leucocyte antigen;
LUVs	large unilamellar vesicles;
MBP	myelin basic protein;
MHC	major histocompatibility complex;
MLVs	multilamellar vesicles;
MOG	myelin oligodendrocytes glycoprotein;
MP	methylprednisolone;
MPS	methylprednisolone hemisuccinate
MS	multiple sclerosis;
OLVs	oligolamellar large vesicles;
PEG	polyethylene glycol;
PHCCC	*N*-Phenyl-7-(hydroxy-imino)cyclopropa[*b*]chromen-1*a*-carboxamide;
PLP	proteolipid protein;
PPMS	primary progressive multiple sclerosis;
PRMS	progressive relapsing multiple sclerosis;
RRMS	relapsing remitting multiple sclerosis;
SPMS	secondary progressive multiple sclerosis;
SUVs	small unilamellar vesicles;
TMEV	Theiler’s murine encephalomyelitis virus;
TMP	tempamine;
